# Confronting heterogeneity in cryogenic electron microscopy data: Innovative strategies and future perspectives with data-driven methods

**DOI:** 10.1016/j.sbi.2024.102815

**Published:** 2024-04-23

**Authors:** Dari Kimanius, Johannes Schwab

**Affiliations:** 1 MRC Laboratory of Molecular Biology, Francis Crick Avenue, Cambridge, CB2 0QH, UK; 2 CZ Imaging Institute, 3400 Bridge Parkway, Redwood City, CA 94065, USA

**Keywords:** Cryo-EM, Representation Learning, Deep Learning, Generalization, Robustness

## Abstract

The surge in the influx of data from cryogenic electron microscopy (cryo-EM) experiments has intensified the demand for robust algorithms capable of autonomously managing structurally heterogeneous datasets. This presents a wealth of exciting opportunities from a data science viewpoint, inspiring the development of numerous innovative, application-specific methods, many of which leverage contemporary data-driven techniques. However, addressing the challenges posed by heterogeneous datasets remains a paramount yet unresolved issue in the field. Here, we explore the subtleties of this challenge and the array of strategies devised to confront it. We pinpoint the shortcomings of existing methodologies and deliberate on prospective avenues for improvement. Specifically, our discussion focuses on strategies to mitigate model overfitting and manage data noise, as well as the effects of constraints, priors, and invariances on the optimization process.

## What is structural heterogeneity and why is it challenging?

The growing popularity of cryogenic electron microscopy (cryo-EM), combined with improvements in microscopy hardware and automated data collection, has led to a surge in data. This underscores the need for quicker, more automated, and reliable data processing [1–3]. In the cryo-EM data processing pipeline, 3D reconstruction, reiterated at multiple stages, typically consumes the most computational time.

The extremely low signal-to-noise ratio (SNR) in cryo-EM data and numerous undetermined parameters make optimization particularly challenging. This is due to the lack of a single well-defined solution, creating an ill-posed optimization problem [4,5].

**Figure 1:**
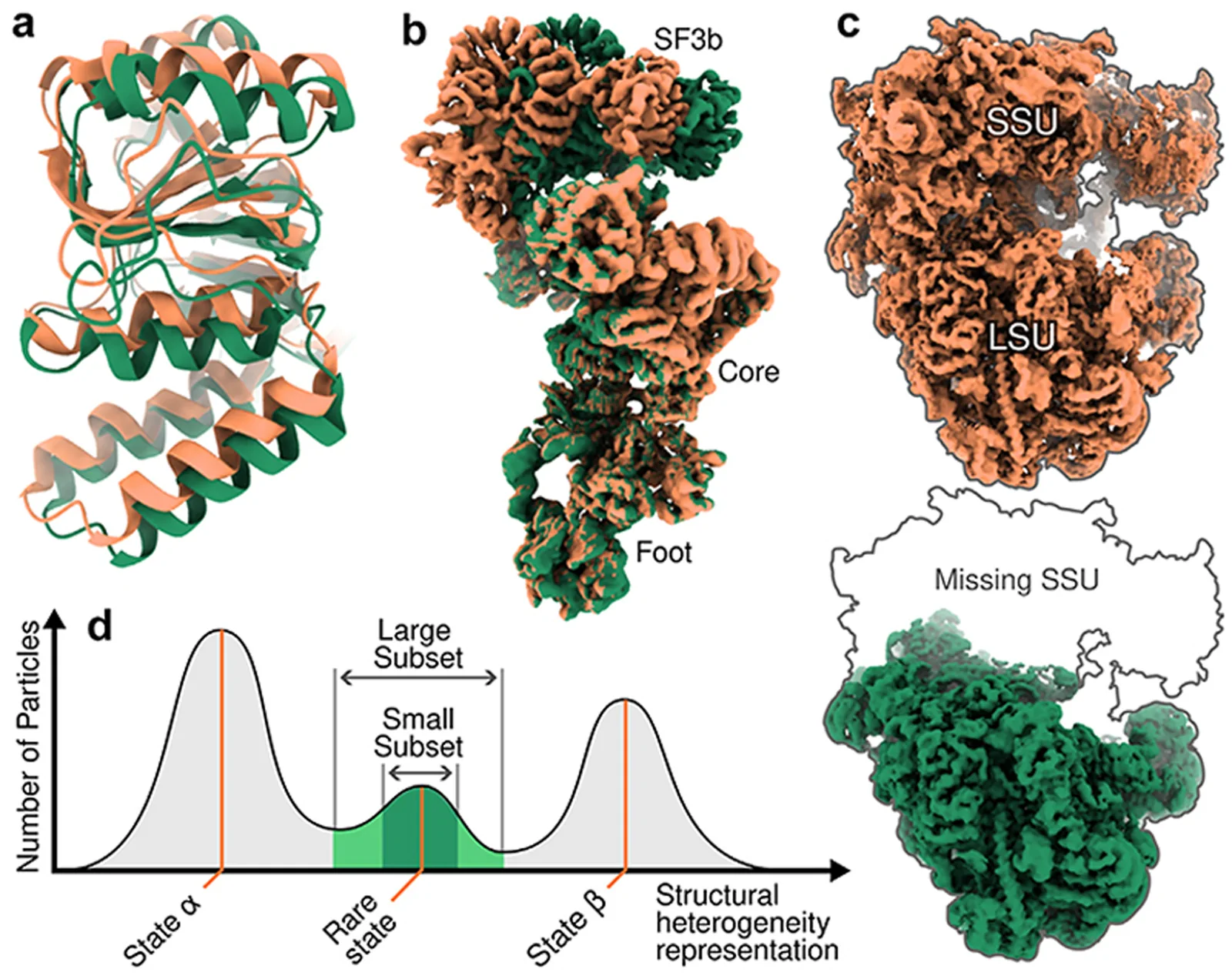
Examples of SH and an illustrative distribution of particle observations in structural HR. **(a)** Displays two superimposed conformational variants of the GroEL subunit as observed in experimental cryo-EM data [8], exemplifying small flexibility visible only at high resolution. Also shown in the graphical abstract. **(b)** Shows two conformational variants of a precatalytic spliceosome [16,17], highlighting the large domain movement of the SF3b subunit. The structure of the spliceosome’s core and foot remains similar in both variants. **(c)** Reconstructions of the Plasmodium falciparum 80S ribosome [18], one missing the small subunit, illustrating compositional heterogeneity. The large subunit remains consistent in both reconstructions. **(d)** A hypothetical one-dimensional HR depiction for a sample with three structural macrostates. To analyze the transient “rare” state, characterized by few particles, two subsets in the HR are selected for refinement. The larger subset, with more particles, typically offers a better SNR, while the smaller subset ensures better structural consistency. The subset yielding the highest resolution reconstruction in the variable region depends on the SH nature, SNR, and the method addressing the SH. cryo-EM, cryogenic electron microscopy HR, heterogeneity representation; SH, structural heterogeneity; SNR, signal-to-noise ratio.

Complexity escalates when considering structural heterogeneity (SH) in particle images. This phenomenon, a fundamental aspect of biological molecules, arises from either conformational or compositional heterogeneity. The former involves samples with identical molecular composition but varying structural conformations, while the latter is due to changes in molecular composition. See Figure 1 for examples and Section The role of constraints and priors for more details.

Current software handles datasets with minimal SH, ensuring robustness. Yet, effectively managing data with significant SH remains a critical challenge, as it is essential for high-resolution 3D reconstructions. Additionally, SH often holds key information about a sample’s biological properties, further necessitating precise analysis [6–9].

Recent developments have led to more expressive models that work on a perparticle 3D volume basis, improving detail representation. However, this increases the number of optimization parameters, which intensifies the ill-posedness problem. Therefore, accurate estimation requires regularization, a key aspect also discussed in Section The role of constraints and priors.

## What is the impact of noise and overfitting?

Due to the ill-posed optimization problem, without proper regularization, errors accumulate in the model, adversely affecting reconstruction results, known as overfitting.

Established 3D classification algorithms explicitly monitor and manage both data and model noise to prevent overfitting [10–12]. This is necessary as each cryo-EM dataset has a unique spectral SNR, influenced by experimental variables like microscopy optics, ice thickness, and the sample’s molecular size, composition, and stability. Additionally, during refinement, factors such as errors in pose estimation, contrast transfer function (CTF), particle count, and the characteristic of the SH affect the spectral SNR of the reconstruction [13–15]. These factors will play a crucial role in the scale of the structural features that can be discerned as well as the scale of SH that can be identified and distinguished.

3D classification algorithms aim to increase signal correlation within subsets by grouping particles with maximum structural uniformity. This increased correlation within subsets should positively impact the reconstruction SNR. However, SNR scales linearly with particle number. Thus, for size-limited datasets, balancing signal consistency and subset size is crucial, typically requiring expert manual supervision and multiple 3D classification and refinement reruns, see Figure 1d. Other approaches, discussed below, can lessen the need for human supervision and enhance data efficiency, which would allow SNR to scale more favorably with particle number.

## Structural heterogeneity representation

Every method designed for examining SH in cryo-EM data necessitates a form of representation to express this heterogeneity, which we define as heterogeneity representation (HR). In essence, similar to pose, it is a perparticle parameter, and it assigns each particle a single 3D structure. As discussed in the previous section, it is yet another optimization parameter and subject to overfitting. The HR of a method can, for example, be a hard class assignment, the coefficients corresponding to a linear combination of densities, or the output of a neural network, often called latent space representation. Whereas the first example is a discrete HR, the rest are continuous representations [19–30].

As previously mentioned, a discrete HR that is used in established 3D classification algorithms has the limitation of severely restricting the expressive power of the model. Small conformational fluctuations and continuous flexibility are beyond the expressive capacity of a discrete model. To address these limitations, numerous methods rely on continuous representations. However, as this increases the parameter space volume, it also increases the room for overfitting. Particularly in deep-learning-based methods, the nonlinear relationship between reconstruction and the HR makes it difficult to estimate the error or overfitting in the learned representation.

In general, methods for SH analysis, basically consist of two ingredients:

1. An algorithm ℱ maps a particle image to its HR. This can be a perparticle optimization problem or inferred by a neural network.
2. An algorithm 𝒢 to obtain the structure, given the HR of a particle image.

Ignoring regularization, in the case of established methods, like 3D classification, the algorithm ℱ can be formalized by the following:

**Linear methods**

For a given image *y*, find the optimal vector *z* = [*z*_1_, *z*_2_, …,*z*_*K*_] such that:

$$\|\frac{1}{\sigma^{2}}\left[y-{𝒞}_{y}P_{\varphi }\left(\sum_{i=1}^{K}z_{i}V_{i}\right)\right]{\|}^{2}\;\text{is}\;\text{minimized}$$

Here, *V*_*i*_ is the set of 3D volumes, *P_φ_* is the projection after alignment with the estimated pose of the particle, 𝒞_*y*_ and *σ*^2^ are the CTF and the power of the noise of *y*.

When evaluated in Fourier space, *σ*^2^ commonly varies significantly as a function of frequencies, making it a crucial factor in cryo-EM. Particularly in 3D classification, the vector *z* is one-hot encoded, which means that it is restricted to only a single one and the rest zero values. The location of the 1-entry corresponds to the class assignment. Algorithm ℱ would simply choose the structure *V* corresponding to the 1-entry.

The GroEL structure, shown in Figure 1a and the graphical abstract, exhibits predominantly discrete SH, which makes it amenable to 3D classification. Although each helical segment undergoes relatively minor conformational shifts, they are interconnected with other displacements within the structure, creating a substantial signal that facilitates the differentiation of various states.

3DVA [31] abandons the one-hot restriction of the solution vector *z*, which gives a new type of algorithm, providing a continuous HR. Note that algorithm ℱ is directly dependent on 𝒢, which changes during the optimization process as the densities *V*_*i*_ or other parameters are refined. Despite the linear representation of the reconstruction itself, linear models can represent non-linear SH [28,21]. Linear models typically enable direct computation of the optimal HR. In nonlinear models, direct solutions are generally infeasible, necessitating the use of other techniques such as iterative optimization.

Recent approaches combine the tasks performed by ℱ and 𝒢 into a single variational optimization problem. One popular method is using a variational autoencoder (VAE), which is designed to learn the HR indirectly through the internal representation of deep neural networks, often referred to as the latent space or latent variables. In this case, ℱ corresponds to an encoder and 𝒢 to a decoder network. Again, ignoring regularization, these methods can be formalized as follows:

**Non-linear methods**

For a given image *y*, find optimal network parameters *θ* and *ϕ* for encoder ℱ_*θ*_ and decoder 𝒢_*ϕ*_ such that for *z* = ℱ_*θ*_(*Y*):

$$\|\frac{1}{\sigma^{2}}\left[y-{𝒞}_{y}P_{\varphi }\left({𝒢}_{\phi }(z)\right)\right]{\|}^{2}\;\text{is}\;\text{minimized}.$$

Both linear and nonlinear methods aim to make ℱ invariant to CTF, pose, and SNR, enhancing sensitivity to SH. Additionally, it’s essential for ℱ to generalize to unseen data not included in the optimized dataset, i.e. the training dataset. Achieving this robustness in nonlinear methods is a known challenge in machine learning [32,33].

Given that noise in cryo-EM images typically vastly exceeds the signal power [34,35], VAE approaches using experimental images as direct network inputs will tend to condition ℱ on data noise rather than structural features. Thus, ℱ in these methods often correctly maps each particle image to its HR mainly through sample image memorization rather than based on features observed in the images. Memorization is a key factor in overfitting within generative models and is notably observed in VAEs, where it negatively impacts convergence and generalizability [36]. This problem is more acute in cryo-EM data due to the extremely low SNR and is primarily reflected in poor generalizability to unseen data, which means that ℱ will map a validation dataset to an incorrect distribution, divergent from that of the training dataset. This discrepancy renders conventional model error assessment via validation datasets ineffective.

Current methods to mitigate overfitting in VAEs, especially under the challenging low SNR conditions of cryo-EM, include limiting network parameters, applying weight decay, and restricting other parameter optimizations, like pose. Additionally, the use of variational inference in VAEs enhances robustness by facilitating a more flexible assignment of the HR, which has an effect akin to marginalization over pose, often used during reconstruction. However, these approaches often fall short as they primarily limit the expressive capacity of the model rather than directly enhancing feature extraction from noisy data. This limitation underscores the urgent need for more advanced feature extraction techniques to limit memorization and improve generalization of the model used for ℱ.

## The role of constraints and priors

Another key design decision in methods addressing SH involves the 3D volumetric representation of reconstructions, used in algorithm 𝒢. We will next explore the algorithmic strengths and weaknesses of these decisions and their potential for effective regularization when estimating SH. Table 1 enumerates several methods along with key aspects for both ℱ and 𝒢.

## Fourier-space methods

Fourier-space methods are here defined as methods that represent and optimize the model parameters in Fourier space. These methods have become the prevalent choice in cryo-EM reconstruction algorithms due to their efficiency in handling the inherent challenges of 3D reconstruction from 2D projections. Fourier-space methods leverage the Fourier-Slice theorem, which enables the conversion of 2D projection images into reciprocal space, where operations like averaging and filtering become more straightforward. This simplifies pose optimization and limits numerical error in the correction of optical aberrations, which fundamentally impact the quality of the reconstruction.

**Figure 2:**
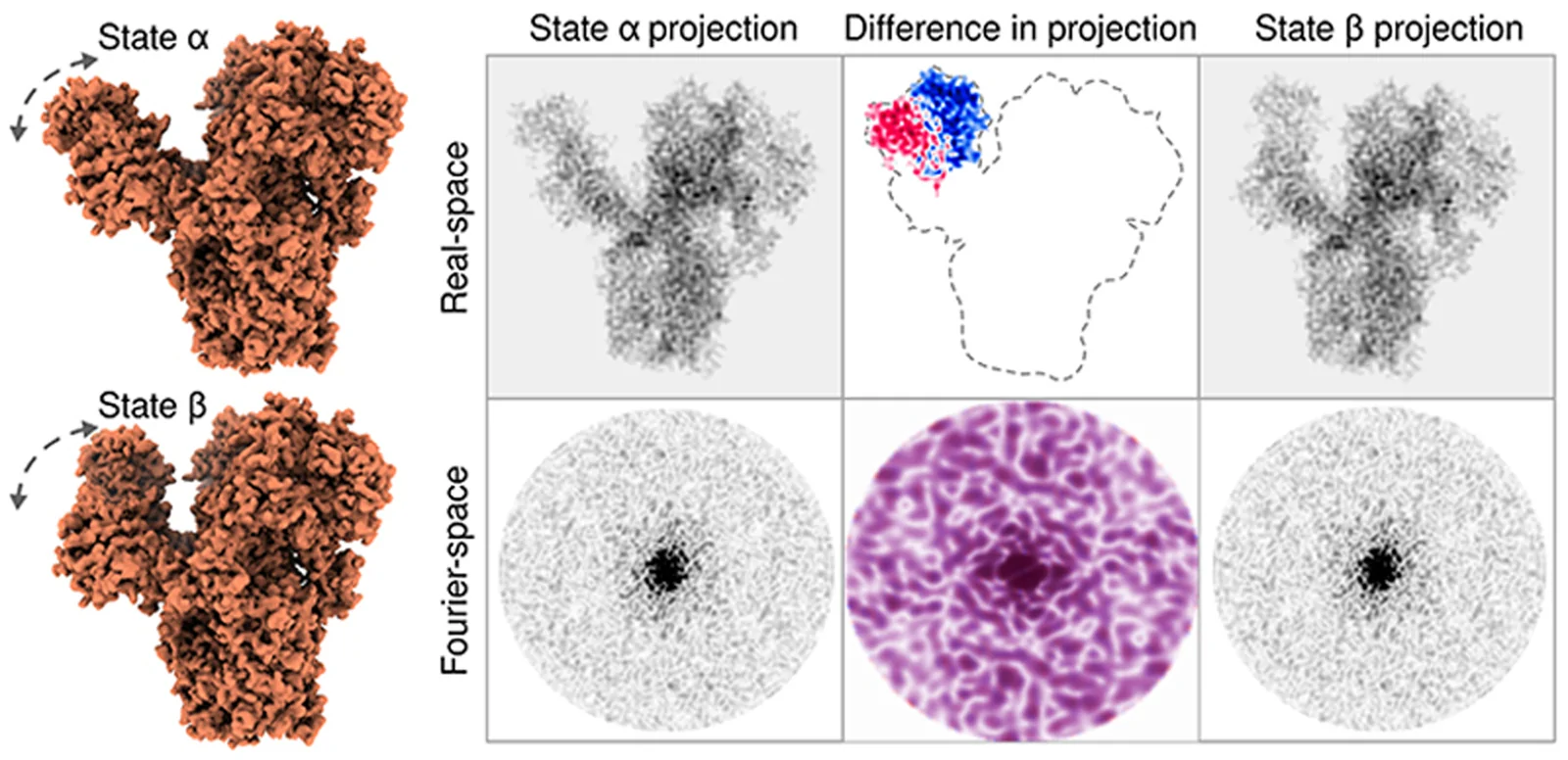
Illustration of local structural motion observed in real-space and Fourier-space projections. The real-space projection’s difference image (middle-top) shows localized changes, while the differences in Fourier coefficients are distributed globally. This illustration is based on simulated data of the coro-navirus spike (E2) protein (https://doi.org/10.5281/zenodo.7182156).

However, the locality of real-space operations, like deformations or masking, cannot be described efficiently in Fourier space (see Figure 2). Therefore, Fourier-space methods are particularly advantageous for analyses where the locality in real space is less important, which includes analyzing compositional heterogeneity.

## Real-space methods

Representing the 3D reconstruction and SH with real-space model parameters offers several benefits: Solvent masks and local filters are applicable directly, bypassing the need for transformations [37,38]. Strong priors, such as neural networks trained to identify and enhance protein-like features, can be effectively utilized [17]. Molecular motion can be directly handled through constraints based on smoothness, local mass conservation, or even molecular geometry or biochemical priors. Additionally, methods that estimate deformations have the potential to improve the reconstruction of a single “ground” state by essentially reversing the deformation of all particles [22–24]. This is especially useful if there are no discrete states or the number of particle images is limited.

Various methods utilize different real-space representations for the 3D volumetric reconstruction and the SH. One strategy involves representing the 3D density as a voxel array and employing tools from partial differential equations, like finite element methods, for the SH. This allows for direct deformation field estimation with local rigidity priors, as shown in Ref. [22]. However, the voxel-based representation is computationally costly due to the need to access the entire 3D grid for each projection operation, so typically the images and volumes have to be downsampled.

To overcome this problem, more efficient basis-function expansions like Gaussian Mixture Models (GMMs) and Zernike polynomials have been suggested. These methods significantly reduce the parameters needed to represent 3D structures. Although GMMs can generally be difficult to optimize, they have demonstrated effective convergence in recent similar applications outside cryo-EM [39]. Particularly, GMMs efficiently maintain high-resolution information. Even when employing a Gaussian per atom, the coefficient count is far lower than in voxel-based representations. This reduction is attributed to the large amount of unmodeled disordered space in 3D cryo-EM reconstructions, often comprising solvents or unstructured lipids in membrane-bound complexes. Furthermore, the fact that the Fourier transform and the projection of a GMM are again a Gaussian functions, comes in handy for numerical efficiency [40,23]. In the Zernike3D approach [24], series expansion is limited to a few coefficients to avoid overfitting, and a penalty term is added for substantial deformations, excluding deformation in the projection direction.

GMMs also facilitate the integration of constraints into an atomic representation. This allows for direct regularization of the SH using strong high-resolution priors, such as an elastic network model. Constraining distances between bound atoms or atom groups, represented by Gaussian, have proven to yield more physically plausible motions [23,25]. Furthermore, this representation can incorporate guidance from coarsegrained Molecular Dynamis (MD) simulations [26,41] or normal mode analysis (NMA) [42,43]. However, as indicated in Ref. [23], caution is necessary when applying such priors in highly flexible regions with low model certainty. In these regions, the use of molecular geometry or biochemical priors can lead to inaccurate deformation assessments and misinterpretations of the data. This is further compounded by the limited ability to validate results, as the same priors often serve for model validation as well.

However, cautious incorporation of stronger priors into reconstruction holds significant promise for enhancing data efficiency and mitigating limitations imposed by SNR dependency on particle number, as detailed in Section What is the impact of noise and overfitting?. For example, local mass conservation priors enable extrapolation of structural information from individual particles to aid in reconstructing adjacent states, rather than exclusively the state observed in each image.

## Conclusions

Recent advancements have addressed several limitations of established algorithms, leading to their broader adoption. Nonetheless, classical algorithms such as 3D classification remain prevalent. We contend that further replacement of these algorithms with newer, more robust solutions is feasible. However, to achieve this, we must overcome specific challenges of these novel solutions, particularly the inadequate handling of model over-fitting, data noise, and the issue of generalization to unseen data, through improvements to invariant feature extraction.

Processing pipelines generally comprise two main stages. The first stage focuses on isolating the target complex by eliminating impurities, empty images, or off-target structures. During the second stage, users explore various methods to enhance the reconstruction’s resolution. Automating most human expert decisions in the first stage is possible, and Fourier-space methods are ideal for this due to their speed. In the second stage, there is a larger need for real-space methods that, as outlined in Section The role of constraints and priors, can better handle conformational heterogeneity, which becomes critical at higher resolutions. This hybrid approach facilitates rapid, automated early-stage processing while leveraging the stronger priors offered by real-space methods.

## Figures and Tables

**Table 1:** Overview of methods for analyzing SH in cryo-EM data. The Space column indicates whether each method primarily uses real or Fourier space for volumetric reconstruction. Although Fourier-space methods allow real-space operations like solvent masking, not all support this; CryoDRGN, due to its nonexplicit volume representation, makes such tasks computationally infeasible. SH-type specifies whether the method is best suited for compositional (com) or conformational (flex) heterogeneity. The Representation column describes the representation of the model quantities (e.g. volumes and deformations), and Regularization describes what type of application-specific explicit prior knowledge is used in this representation. Some methods include implicit regularization by volumetric/deformation repre-sentations or by network architecture. The abbreviation lin. com. is for linear combination. Note that this table aims to primarily list methods with a diverse set of design choices. Please refer to Ref. [44] for a more comprehensive overview.

Method	Space	Representation	SH-type	Regularization
3D-classification [10,11]	Fourier	Discrete volumes	Com	*L* ^2^
3DVA [31]	Fourier	lin. com. of volumes	Both	–
Multibody [30]	Fourier	Rigid body deformation	Flex	*L* ^2^
CryoDrgn [19]	Fourier	Per particle volume	Both	–
SFB [21]	Fourier	lin. com. of volumes	Both	–
e2gmm [20,40]	Real	Gaussian	Both	–
DynaMight [23]	Real	Gaussian displacement	Flex	Local distances
3DFlex [22]	Real	Deformation (grid)	Flex	Rigidity
Zernike3D [24]	Real	Deformation (Zernike)	Flex	Amount &directional
MDSPACE [26,41]	Real	Gaussian	Flex	MD-Simulation
(Deep)-HEMNMA [42,43]	Real	Gaussian	Flex	NMA
CryoStar [25]	Real	Gaussian &volume	Flex	Local distances

## Data Availability

All data used in this paper are already publicly available on online databases.
